# Case Report: Dupilumab Successfully Controls Severe Eczema in a Child With Elevated IgE Levels and Recurrent Skin Infections

**DOI:** 10.3389/fped.2021.646997

**Published:** 2021-09-29

**Authors:** Tejas P. Joshi, Sara Anvari, Meera R. Gupta, Carla M. Davis, Joud Hajjar

**Affiliations:** ^1^Baylor College of Medicine, Houston, TX, United States; ^2^Department of Pediatrics, Baylor College of Medicine, Houston, TX, United States; ^3^Wiliam T Shearer Center for Human Immunobiology, Texas Children's Hospital, Houston, TX, United States

**Keywords:** hyper-IgE syndrome, dupilumab, severe eczema, primary immunodeficencies, pediatrics-children

## Abstract

The efficacy of dupilumab in pediatric patients with severe eczema presenting in the setting of elevated immunoglobulin E (IgE) levels and recurrent bacterial skin infections is not well-understood. Here we present the case of a child with elevated IgE levels in whom dupilumab treatment led to remarkable control of his eczema and recurrent skin infections. We also review the use of dupilumab in other patients with molecularly proven cases of hyper IgE (HIGE) syndrome. Our case supports the notion that dupilumab may have a seminal application in treating severe eczema that occurs in the setting of elevated IgE levels and recurrent bacterial skin infections.

## Introduction

Management of severe atopic dermatitis presenting in the context of elevated immunoglobulin E (IgE) levels and recurrent bacterial skin infections is challenging. Dupilumab is an interleukin (IL)-4Rα antagonist that is Food and Drug Administration (FDA) approved to treat moderate to severe asthma (1) as well as severe atopic dermatitis recalcitrant to topical treatments (2). However, there is a paucity of clinical knowledge regarding the effectiveness of dupilumab in treating severe eczema that presents in the setting of elevated IgE levels and recurrent bacterial skin infections. Here, we report the case of a patient with severe eczema, elevated IgE levels, and recurrent bacterial skin infections who experienced significant improvement of his atopic dermatitis, recurrent skin infections, and quality of life following treatment with dupilumab.

We also note that our case results may be applicable in the management of hyper-IgE (HIGE) syndromes. Hyper-IgE (HIGE) syndromes are rare inborn errors of immunity (IEI) marked by severe atopic dermatitis, recurrent skin infections, and elevated IgE levels (3). Currently, there exists no paradigm of treatment for HIGE syndrome, with the management of HIGE largely centering on scrupulous skin care and antibiotics aimed at treating infections and preventing long-term infectious complications (4, 5). While our patient does not represent a case of classic HIGE syndrome, the regression of our patient's severe eczema, along with the drop in IgE levels and halting of recurrent infections, suggests that dupilumab may have a potential application in patients presenting with similar clinical dilemmas.

## Case Report

A currently 13-year-old Caucasian male was evaluated in the Allergy and Immunology clinic at age 8 years for severe eczema, asthma, and multiple food allergies. He had a normal birth history and normal development. He developed eczema in infancy, which became severe by the age of 8 years. Despite excellent skincare, including wet wraps, topical steroids, and antibiotics, the patient developed multiple *Staphylococcus aureus* skin infections, requiring both topical and systemic antibiotics in addition to systemic steroids. His scoring atopic dermatitis (SCORAD) score upon initial evaluation was 91.7/103. In addition to his eczema, he has severe asthma, allergic rhinitis, and multiple IgE-mediated food allergies, including eggs, peanut, and tree nuts. He also had drug allergies to multiple antibiotics with reactions ranging from rash to hives and angioedema. He had delayed primary teeth shedding; eight teeth needed extraction by a dentist and were described to be soft with hypercalcification. He had one fracture of his left wrist due to trauma after falling on his arm. There is no known interstitial lung disease, and a chest X-ray done at age 11 was normal. His family history was significant for eczema and delayed primary teeth shedding in a single tooth in his mother, who became completely asymptomatic in adulthood. No consanguinity and no other known family member with diseases or symptoms that might be relevant to his presentation were reported by the patient's family.

The initial immune evaluation showed persistently elevated IgE. His IgE started around 20,000 KU/L at age 11, and persistently remained >50,000 KU/L since age 12, even when he did not have an active skin infection. He had normal IgG/A/M, and normal responses to protein and polysaccharide-based vaccines (Table 1). He had occasional lymphocytosis but normal distribution of lymphocyte subsets (CD3^+^/CD4^+^/CD8^+^/CD19^+^/ and CD3^−^CD16^+^CD56^+^) and normal lymphocyte proliferation to mitogens (phytohemagglutinin, concanavalin A, pokeweed mitogen) and recall antigens (*Candida*, tetanus, and diphtheria). He had low normal CD4 T^+^ cell induction of intracellular IL-17 with stimulation (Table 2). Chromosomal Microarray Analysis (CMA) showed a gain of 16q11.2 and a gain of 9p24.3 involving the dedicator of cytokinesis 8 (*DOCK8*) gene, of unclear significance. However, DOCK8 protein expression (flow cytometry after permeabilization and staining with DOCK8-specific monoclonal antibody) on peripheral blood mononuclear cells was normal (Seattle Children's Immunology Diagnostic Lab). Additionally, clinical trio exome sequencing (Baylor Genetics) revealed a heterozygous c.7339C>T (p.Arg2447Ter) change in the Filaggrin *(FLG)* gene inherited from the father [Minor Alle Frequency MAF was 5/3,733, reported using e Exome Variant Server (EVS)]. Of note, the father has allergic rhinitis but no atopic dermatitis.

**Table 1:** Humoral immunity panel: patient had elevated IgE levels but normal IgG/M/A and normal responses to protein and polysaccharide-based vaccines.

	**Before treatment**	**After treatment**	**Reference values**
IgE	>50,000 kU/L	5,820 kU/L	<200 kU/L
IgG	1,100 mg/dL	1,170 mg/dL	641–1,353 mg/dL
IgM	70 mg/dL	71 mg/dL	40.0–180.0 mg/dL
IgA	97 mg/dL	109 mg/dL	66.0–295.0 mg/dL
Absolute eosinophil count	3,081 cells/uL	190 cells/uL	
*Streptococcus pneumonia*	17/23 Serotypes protective	—	
Tetanus,Diphtheria,Candida	Protective	—	

*The patient showed markedly reduced IgE levels following treatment. IgE, immunoglobulin E; IgG, immunoglobulin G; IgM, immunoglobulin M; IgA, immunoglobulin A*.

**Table 2:** Treatment with dupilumab led to normalization of CD4 T cell induction of intracellular IL-17 following stimulation.

**Unstimulated**	**% Positive**	**Stimulated**	**% Positive**	**Reference range (%)**
**Before treatment**				
CD4+IL17a+	0	CD4+IL17a+	0.4	≥0.5
**After treatment**				
CD4+IL17a+	0.02	CD4+IL17a+	0.63	≥0.5

At age 8, he was started on cyclosporine 2–3 mg/kg and oral trimethoprim/sulfamethoxazole in an attempt to control his eczema. Nevertheless, he continued to develop severe eczematous flares, leading to recurrent superimposed bacterial infections requiring hospitalizations and intravenous antimicrobial agents. At age 10, he developed herpes simplex virus (HSV) skin infections and recurrent generalized lymphadenopathy. Lymph node biopsy during one of the flares was negative for malignancy, but cultures grew methicillin-resistant *Staphylococcus aureus*. He also experienced vernal keratoconjunctivitis of both eyes, with significant epitheliopathy and corneal irritation, requiring amniotic membrane transplantation and subconjunctival corticosteroid injection, complicated by increased intraocular pressure following steroid injection.

In an attempt to control his severe eczema, he received two doses of omalizumab 300 mg at age 10, which provided some skin improvement. Still, the medication had to be discontinued due to developing anaphylaxis (laryngeal swelling and wheezing) shortly after the second dose. Methotrexate was added with no clinical response. Given his severe eczema, recurrent infections, and the loss of school days, and failure of all other treatments, he was started on intravenous immunoglobulin (IVIG) 1 g/kg in an attempt to modulate his eczema and prevent recurrent infections. IVIG helped decrease the frequency of infections and recurrent lymphadenitis, but he continued to have severe eczema. Treatment consisting of hydrocortisone 2.5% and triamcinolone 0.1% ointments was also unable to ameliorate eczema to any significant extent.

**Figure 1:**
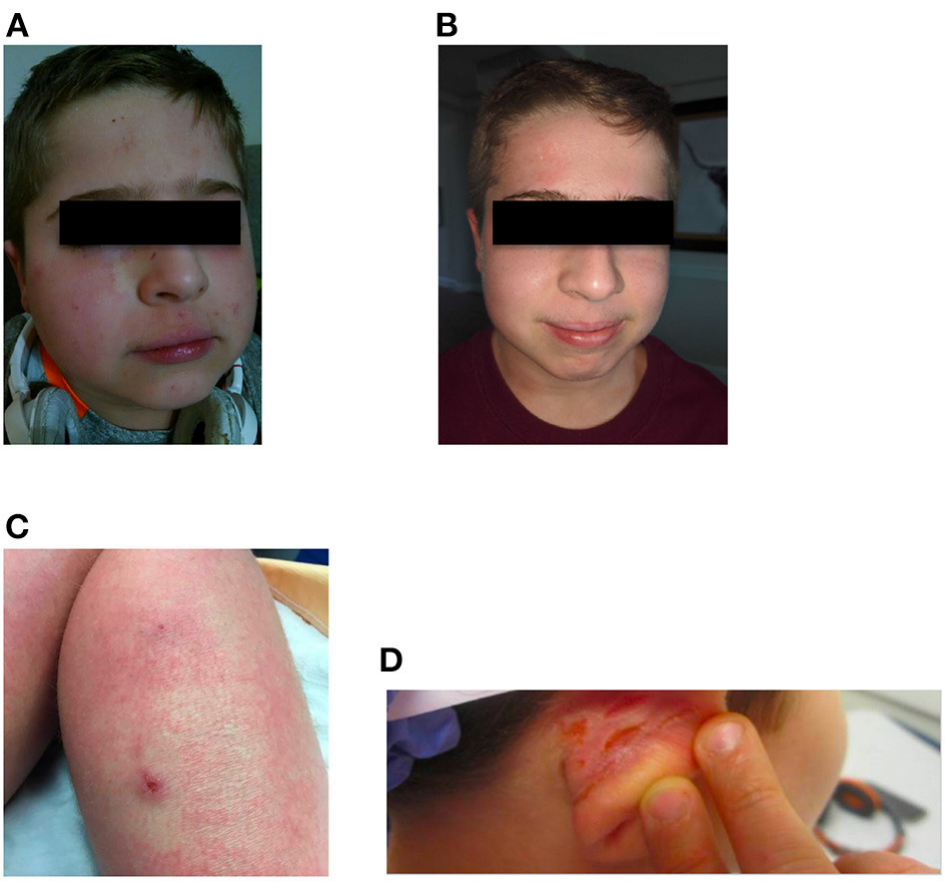
Facial eczema prior to **(A)** and after **(B)** starting treatment with dupilumab. **(C,D)** Show lesions on the right leg and right ear, respectively, prior to dupilumab treatment. Although corresponding images for **(C,D)** after treatment were not captured, the lesions seen prior to treatment resolved after dupilumab treatment.

At age 12, the patient was started on dupilumab 200 mg subcutaneously every 2 weeks, which led to a considerable improvement in his eczema, with his SCORAD score dropping to 23.95/103. The patient experienced a marked reduction in pruritus and erythema. The control of his eczema was accompanied by a significant decrease in skin infections. Furthermore, the patient was able to discontinue topical and systemic steroids (Figure 1). His keratoconjunctivitis significantly improved. His overall quality of life was also significantly improved, and the patient was able to return to school full time and travel on family vacations.

**Figure 2:**
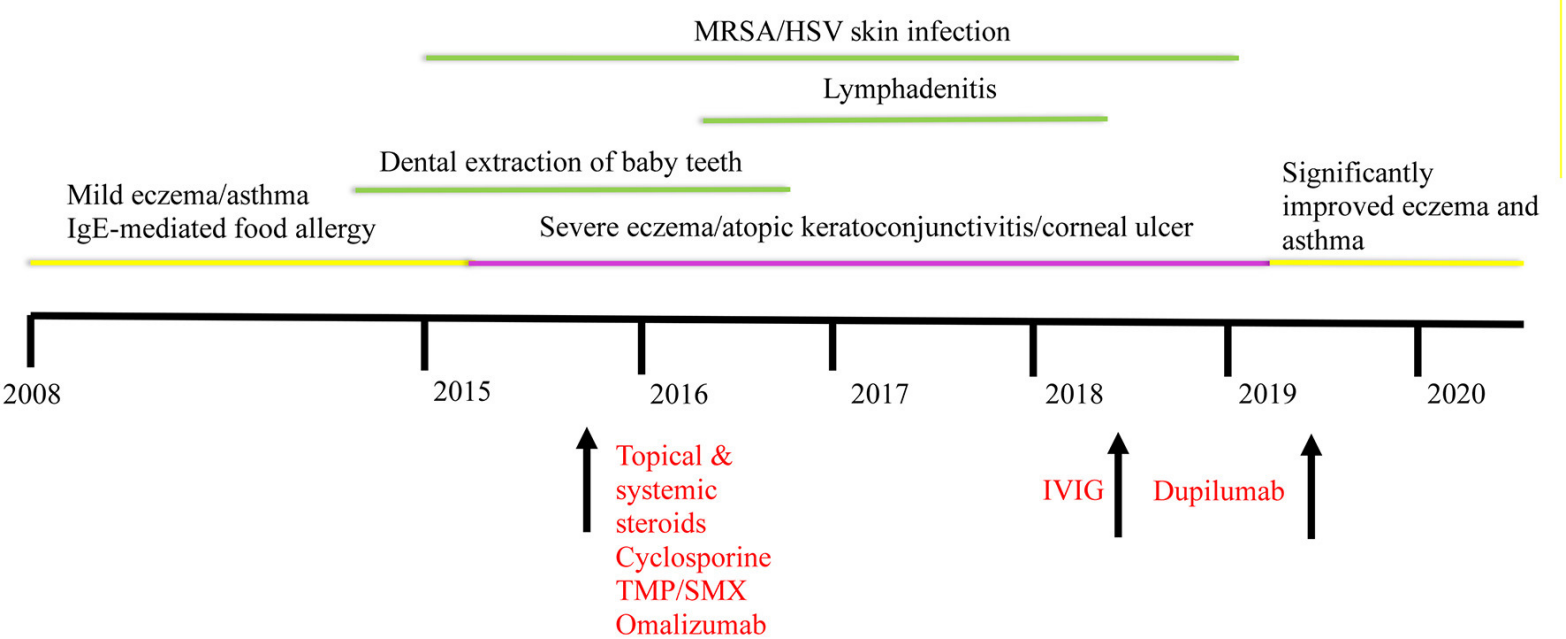
Timeline illustrating the development of our patient's clinical phenotype and improvement following dupilumab. Interventions are indicated in red. HSV, herpes simplex virus; IgE, immunoglobulin E; IVIG, intravenous immunoglobulin; MRSA, methicillin resistant *Staphylococcus aureus*; TMP/SMX, trimethoprim/sulfamethoxazole.

Additionally, following treatment with dupilumab, the patient's IgE levels decreased to 5,820 IU/mL. Repeat immune evaluation showed complete normalization of CD4 T cell induction of intracellular IL-17 with stimulation (Table 2). An attempt to discontinue IVIG while on dupilumab led to worsening of his eczema, and thus the patient has remained on IVIG. However, given the burden of immunoglobulin treatment on the child and family, a second attempt for stopping IVIG was successful, and the patient is currently off IVIG. He continues to require cyclosporine 2 mg/kg to avoid itching and redness of the skin. However, we will attempt weaning cyclosporine if eczema continues to be well-controlled. A timeline illustrating the development of our patient's clinical phenotype and treatment is summarized in Figure 2.

In addition to his excellent eczema control, his asthma became well-controlled. Prior to treatment with dupilumab, the patient had to utilize his albuterol inhaler at least once a week. Following treatment, the patient denied having to resort to his rescue albuterol inhaler. Moreover, his height improved from the 10th percentile to the 25th percentile, and his weight dropped from the 80th percentile to the 50th percentile. The change in the growth pattern could be partially attributed to decreased systemic steroid use. In addition, with his skin improvement, he felt more comfortable participating in exercise. Regarding food allergies, the patient continues to avoid all food allergens; we plan to re-evaluate food and medication allergies once pandemic restrictions are lifted. Altogether, dupilumab led to remarkable control of the patient's eczema, halted recurrence of bacterial skin infections, and led to a precipitous drop in the patient's IgE levels. Moreover, the steroid-sparing effect improved the patient's symptoms, quality of life, and growth. Nineteen months after starting treatment, he continues to tolerate dupilumab, with no side effects reported.

## Discussion

Dupilumab is a monoclonal antibody that inhibits IL-4 signaling via the Type I receptor and both IL-4 and IL-13 signaling through the Type II receptor resulting in decreased IL-4 and IL-13 cytokine-induced responses, including the release of proinflammatory cytokines, chemokines, and IgE. Additionally, dupilumab down-regulates many genes salient for epidermal hyperplasia and dendritic and T cell activity, while up-regulating genes important for enforcing epithelial integrity (6). It is currently approved by the FDA for the treatment of moderate to severe atopic dermatitis and asthma (7).

Our patient had a history of severe vernal keratoconjunctivitis of both eyes before starting dupilumab treatment. However, he did not experience any exacerbation of keratoconjunctivitis following dupilumab treatment. Dupilumab-associated conjunctivitis is seen in about 10–20% of patients (8). The etiology is not well-understood, but the risk is increased in patients with severe atopic dermatitis and history of keratoconjunctivitis (9). However, patients who responded well to dupilumab had reduced incidence of conjunctivitis (8, 9). Although our patient had the relevant risk factors to develop dupilumab-associated conjunctivitis, his excellent response to dupilumab might have helped in decreasing his risk for this complication.

The clinical genetic work-up for our patient did not identify a definite molecular diagnosis, and further workup is underway to identify a definitive molecular diagnosis. However, the regression of our patient's severe eczema, along with the drop in IgE levels and halting of recurrent infections, suggests that dupilumab may have a potential application in patients presenting with immune dysregulation resulting in severe eczema, elevated IgE levels, and recurrent bacterial skin infections. As severe eczema, elevated IgE levels, and recurrent bacterial skin infections are also hallmarks of HIGE syndromes, we note that dupilumab may be of utility in the management of HIGE syndromes.

In our literature review, we found two adult patients and three pediatric patients with HIGE syndrome where dupilumab was successfully used to treat eczema (Table 3). Sogkas et al. (10) reported a case of HIGE due to a *STAT3* loss of function in which the patient's eczema regressed remarkably following treatment with dupilumab. Furthermore, the patient developed no further skin abscesses, and their SCORAD values markedly decreased following dupilumab treatment. Impressively, the patient did not experience any side effects, and the results of dupilumab were persistent at a follow-up period of 8 months. Lévy et al. (11) reported an adult case of HIGE due to a *ZNF341* deficiency. Overall, the patient had a significant improvement in their skin with a corresponding decrease in their SCOARD score. No skin infections or side effects were noted, and treatment was well-tolerated. Moraczewski et al. reported the case of a 13-year-old female with HIGE syndrome due to *STAT3* loss of function in whom dupilumab led to improvement of her scalp infections and axillary lymphadenitis (12). Diaz-Cabrera et al. reported two pediatric patients with HIGE syndrome due to *CARD11* loss of function; in both patients, dupilumab led to improvement of eczema and other atopic features, such as asthma, allergic rhinitis, and food sensitivities (12).

**Table 3:** Review of literature regarding patients with HIGE syndrome treated with dupilumab to control refractory eczema.

**Case**	**Age**	**Sex**	**Diagnosis**	**History of infections**	**Other treatments**	**IgE before dupilumab treatment(IU/mL)**	**IgE after dupilumab treatment (IU/mL)**
Diaz-Cabrera et al. (12)	11	F	*CARD11* loss of function	Upper and lower respiratory tract infections; skin infections	None reported	Not reported	Not reported
Diaz-Cabrera et al. (12)	12	F	*CARD11* loss of function	HPV; MCV; upper respiratory tract infections	None reported	Not reported	Not reported
Lévy et al. (11)	48	F	*ZNF341* deficiency	None reported	Class I topical corticosteroids and topical tacrolimus	22,500	7,500
Moraczewski et al. (12)	13	F	*STAT3* loss of function	Bacterial pneumonias complicated by pneumatoceles; recurrent skin abscesses; otitis media	IVIG; doxycycline; culture directed antimicrobial and antifungal therapy	14,000	Not reported
Sogkas et al. (10)	33	F	*STAT3* loss of function	Cutaneous abscesses	UV phototherapy; topical corticosteroids; topical pimecrolimus; co-trimoxazole	12,000	7,500
Our case	12	M	Severe atopic dermatitis, recurrent bacterial skin infections, and elevated IgE levels	*Staphylococcus aureus* and HSV skin infections	Topical and systemic steroids and antibiotics; IVIG; cyclosporine	15,000	5,820

*HIGE, hyper immunoglobulin E; ZNF341, zinc finger protein 341; STAT3, signal transducer and activator of transcription 3; HPV, human papillomavirus; HSV, herpes simplex virus; MCV, molluscum contagiosum virus; IgE, immunoglobulin E; IVIG, intravenous immunoglobulin; F, Female; M, Male*.

In summary, we describe a case of a child with severe eczema, elevated IgE levels, and recurrent bacterial skin infections who had significant clinical improvement in his eczema and asthma following dupilumab therapy. Our case supports the notion that dupilumab may have a seminal application in treating atopic dermatitis arising in the context of recurrent bacterial skin infections and elevated IgE levels. Furthermore, in conjunction with existing literature describing the efficacy of dupilumab for management of HIGE syndrome, our case also suggests that dupilumab may constitute an effective therapy for patients with HIGE syndrome.

## Data Availability Statement

The original contributions presented in the study are included in the article/supplementary material, further inquiries can be directed to the corresponding author/s.

## Ethics Statement

Written informed consent was obtained from the minor(s)' legal guardian/next of kin for the publication of any potentially identifiable images or data included in this article.

## Author Contributions

JH was responsible for the conception and design of the study. TJ wrote the first draft of the manuscript. JH wrote sections of the manuscript. All authors contributed to manuscript revision, read, and approved the submitted version.

## Conflict of Interest

The authors declare that the research was conducted in the absence of any commercial or financial relationships that could be construed as a potential conflict of interest.

## Publisher's Note

All claims expressed in this article are solely those of the authors and do not necessarily represent those of their affiliated organizations, or those of the publisher, the editors and the reviewers. Any product that may be evaluated in this article, or claim that may be made by its manufacturer, is not guaranteed or endorsed by the publisher.
